# Design and Synthesis of Near-infrared Fluorescent Probes for Imaging of Biological Nitroxyl

**DOI:** 10.1038/srep16979

**Published:** 2015-11-20

**Authors:** Yi Tan, Ruochuan Liu, Huatang Zhang, Raoul Peltier, Yun-Wah Lam, Qing Zhu, Yi Hu, Hongyan Sun

**Affiliations:** 1 Key Laboratory of Biochip Technology, Biotech and Health Centre, Shenzhen Research Institute of City University of Hong Kong, Shenzhen 518057, China; 2 Department of Biology and Chemistry, City University of Hong Kong, 83 Tat Chee Avenue, Hong Kong, China; 3 Insitute of Bioengineering, Zhejiang University of Technology, Chaowang Road 18, Hangzhou 310014, China; 4 CAS Key Laboratory for Biomedical Effects of Nanomaterials and Nanosafety, CAS Key Lab of Nuclear Radiation and Nuclear Energy Technology, Center for Multidisciplinary Research, Institute of High Energy Physics, Chinese Academy of Sciences (CAS), Beijing 100049, China

## Abstract

Nitroxyl (HNO), the reduced and protonated form of nitric oxide (NO), has recently been identified as an interesting and important signaling molecule in biological systems. However, research on its biosynthesis and bioactivities are hampered by the lack of versatile HNO detection methods applicable to living cells. In this report, two new near-infrared (NIR) probes were designed and synthesized for HNO imaging in living cells. One of the probes was found to display high sensitivity towards HNO, with up to 67-fold of fluorescence increment after reaction with HNO. The detection limit was determined to be as low as 0.043 μM. The probe displayed high selectivity towards HNO over other biologically related species including metal ions, reactive oxygen species, reactive nitrogen species and reactive sulfur species. Furthermore, the probe was shown to be suitable for imaging of exogenous and endogenous HNO in living cells. Interestingly, the probe was found to be mainly localized in lysosomes. We envision that the new NIR probe described here will serve as a useful tool for further elucidation of the intricate roles of HNO in living cells.

In the past two decades, nitric oxide has been established as an important signaling molecule involved in the regulation of many physiological and pathological processes, including vasodilatation, neurotransmission and immune response^1^,^2^,^3^,^4^. Recently, its one-electron reduced or protonated counterpart, nitroxyl, has been shown to play a distinct but also important biological role^5^,^6^,^7^,^8^. Differences in the biological functions of HNO are attributed to its unique chemical reactivity^9^,^10^. For example, most of the cellular targets of HNO are found to be thiols, metals and metalloproteins. In addition, HNO was shown to react with oxygen to afford peroxynitrite (ONOO^−^). HNO can also react with NO to produce N_2_O_2_^−^, which is a strong oxidant^11^.

Since its discovery, HNO has attracted growing interest in a wide range of applications, ranging from cardiovascular regulation^12^,^13^ to immune system regulation^14^ and neuronal physiology^15^. For instance, HNO has been shown to act as a vasodilator and is therefore considered as a potential therapeutic agent for the treatment of heart failure^16^. As an inhibitor of aldehyde dehydrogenase, HNO also shows potential uses in the treatment of alcohol abuse^17^.

Despite these exciting progress, the detailed biological functions of HNO and its mechanisms of endogenous generation remain unclear. This is largely due to a lack of efficient methods to detect HNO. The molecule is highly reactive and readily dimerizes and dehydrates to nitrous oxide (N_2_O), creating difficulties in accurately detecting it in biological samples^18^. The development of novel chemosensors that allow for both selective and sensitive detection of HNO in biological environments is therefore of great interest.

Previously developed methods for HNO detection include mass spectroscopy^19^, colorimetry^20^, electrochemical detection^21^ and high-performance liquid chromatography^22^. Compared with these methods, fluorescent probes offer numerous advantages. They are simple to use, non-invasive and provide well-defined spatiotemporal resolutions as well as excellent sensitivity, making them ideal candidates for detecting HNO in complex biological environment. A number of fluorescent probes responsive to HNO have been designed. The majority of these probes rely on the reduction of either Cu(II) to Cu(I)^23^,^24^,^25^ or nitroxide to hydroxylamine^26^. Such probes were able to detect HNO in living cells but with various drawbacks. They can be interfered by other biological reductants such as ascorbate or glutathione, limiting their wide applications in biological studies. An alternative HNO probe is designed by taking advantage of the reaction between HNO and triaryl-phosphine. The reaction will yield the aza-ylide intermediate and lead to subsequent ester aminolysis^27^,^28^. A number of fluorescent probes were designed based on this concept. They have shown good selectivity even in the presence of other cellular reductants^29^,^30^,^31^,^32^. The use of these reductant-resistant probes, however, is limited by their short wavelength of excitation and emission.

NIR probes boast two distinct advantages over classic fluorescent probes. First, the low absorption of biological molecules in the NIR region leads to dramatically reduced levels of autofluorescence. Second, NIR light penetrates deeper into biological tissues than visible light, therefore allowing for imaging of deeper tissues structure^33^,^34^. Due to the above prominent properties, NIR probes have become increasingly popular tools in the field of bioimaging. Recently, Rivera-Fuentes and Lippard’s group developed a NIR probe based on the reduction of Cu (II) to Cu (I). However, the probe is not sensitive and the fluorescence increment is only 5-fold^35^. In another example, Chen and his coworkers have developed a NIR probe based on aza-BODIPY to detect HNO^36^. It is noted that the probe required high amount of surfactant, which might cause cell toxicity and affect cell imaging studies. In this report, we have designed and synthesized two new NIR fluorescent probes based on the use of dihydroxanthene (DHX) to detect HNO in cells. Our probes can detect HNO in PBS buffer and do not require the usage of any surfactant or organic solvent. One of the designed probes displayed excellent sensitivity and selectivity towards HNO. The probe was further applied for imaging exogenous and endogenous HNO in living cells.

## Results and Discussion

### Design and synthesis of NIR probes

**Figure 1:**
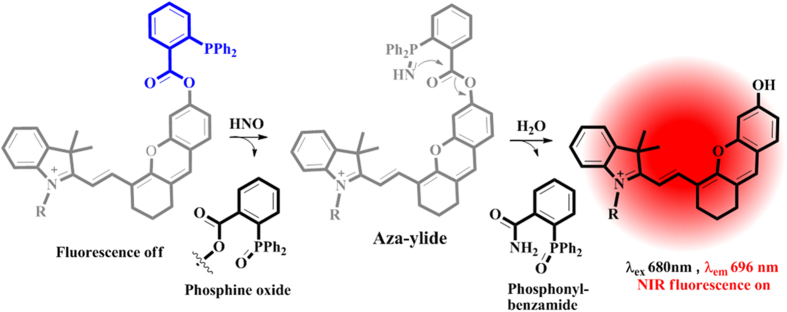
Proposed reaction mechanism of NIR probes reacting with HNO.

The probe consists of two parts: a NIR fluorophore and a HNO reaction moiety (Fig. 1). In our design, DHX was selected as the fluorophore because it has excellent quantum yield and good cell permeability. The probe itself is non-fluorescent, as the DHX fluorophore is protected with triaryl-phosphine moiety. When the probe reacts with HNO, it generates an aza-ylide intermediate that is unstable and will further react with the adjacent ester bond, leading to ester aminolysis and “turn on” of the fluorescence.

**Figure 2:**
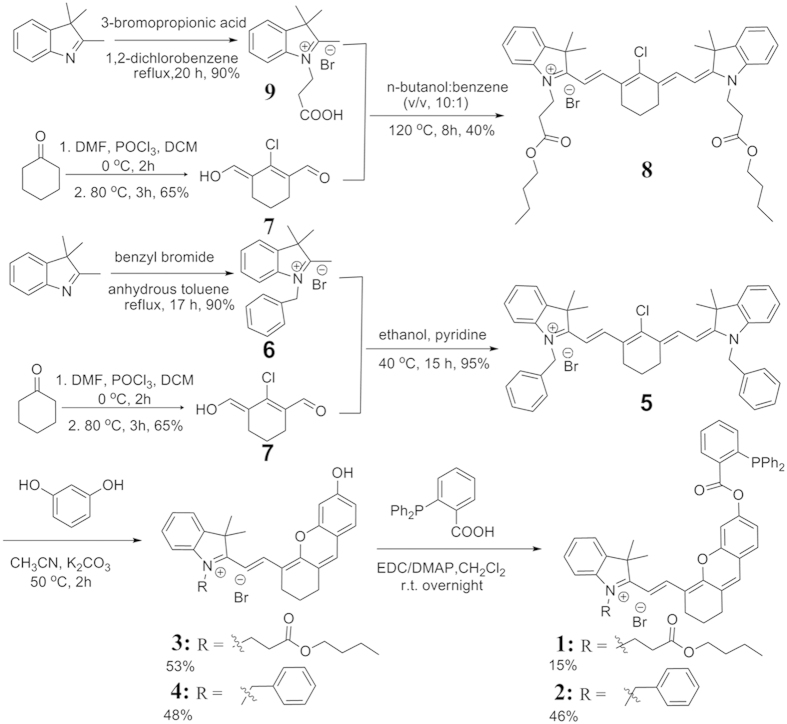
Schematic synthesis of probe (1,2).

A total of two NIR probes were designed and synthesized in our study. As shown in Fig. 2, the R group at nitrogen position of indole is substituted with a lipophilic chain and a benzyl group respectively in probe **1** and **2**. We reckon that different substitutions at nitrogen position of indole moiety may affect the photophysical properties of the probe. The heptamethine chloride compounds **5** and **8** were prepared according to a modified literature method. Compounds **3** and **4** were obtained via retro-Knoevenagel reaction by reacting compounds **8** and **5** with resorcin in the presence of K_2_CO_3_ at 50 °C for 2 h using acetonitrile as the solvent. Further treatment of compound **3** and **4** with 2-(diphenylphosphino)benzoic acid in CH_2_Cl_2_ containing EDCI and DMAP gave the esterification product probe **1** and **2**. After the synthesis, the compounds were characterized with ESI-MS, ^1^H NMR and ^13^C NMR respectively. Details of the synthesis can be found in the Supporting Information.

With the two probes in hand, we first performed preliminary experiments to examine the fluorescent “turn on” properties with and without treatment of HNO (Angeli’s salt is used as HNO donor)^37^. Experiment results showed that the “turn on” effect of probe **1** is superior to that of probe **2.** We then examined the fluorescence property of the two NIR fluorophores **3** and **4** respectively. As expected, the fluorescence intensity of **3** is indeed stronger than that of **4** under the same conditions (Supplementary Fig. S1). Consequently we chose to use probe **1** for the subsequent experiments.

### Photophysical properties of probe 1

**Figure 3:**
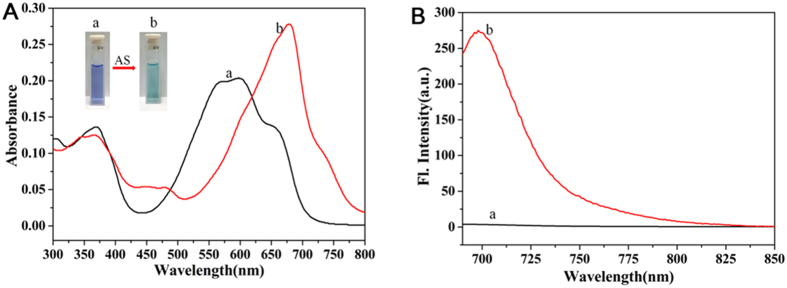
(**A**) UV absorption spectra and (**B**) fluorescence emission spectra (λ_ex_ = 680 nm) of probe **1** (10 μM) in PBS buffer (10 mM, 1% DMSO, pH = 7.4) in the absence (a, black) or the presence (b, red) of 100 μM AS.

The photophysical properties of probe **1** were characterized in PBS buffer in the presence or the absence of Angeli’s salt (AS). As shown in Fig. 3A, probe **1** in PBS solution showed an absorption maximum at 610 nm. Upon addition of AS (100 μM), the absorbance band shifted significantly towards the red wavelengths with a new absorption band observed at around 680 nm. The UV spectrum of the probe after reacting with HNO was similar to that of compound **3** (Supplementary Fig. S4). To the naked eye, this red-wavelength shift translated into a change of the solution color from purple to cyan within only a few minutes of AS addition. Next, fluorescence studies showed that the probe itself emits negligible fluorescence in PBS buffer. After reacting with HNO, the fluorescence signal of probe **1** increased by 67-fold, with a maximum emission intensity at 696 nm (Fig. 3B).

### Detection limit studies

**Figure 4:**
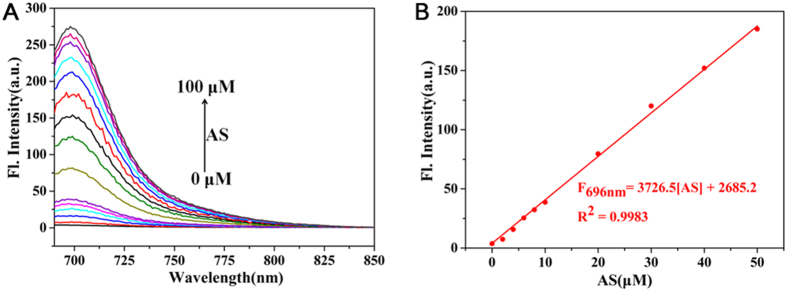
(**A**) Fluorescence spectra of probe **1** (10 μM) in the presence of various concentrations of AS, λ_ex_ = 680 nm, λ_em_ = 690 nm–850 nm. (**B**) Relationship between the relative fluorescence intensity at 696 nm and concentrations of AS (0, 2, 4, 6, 8, 10, 20, 30, 40, 50 μM). Fluorescence spectra were acquired in PBS buffer solution (10 mM, 1% DMSO, pH 7.4).

In order to determine the detection limit of the probe **1**, a detailed titration experiment was performed by recording the fluorescent spectra of **1** in the presence of various concentrations of AS, ranging from 0 to 100 μM. As expected, the fluorescence intensity at 696 nm was found to increase along with AS concentration (Fig. 4A). A linear relationship was established between the fluorescence intensity and AS concentration ranging from 0 to 50 μM, along with a regression equation of F_696nm_ = 3726.5 × [AS] + 2685.2 and a coefficient R^2^ = 0.9983. The detection limit was calculated to be 0.043 μM using the 3σ/s method, indicating the high sensitivity of probe **1**.

### Selectivity studies

**Figure 5:**
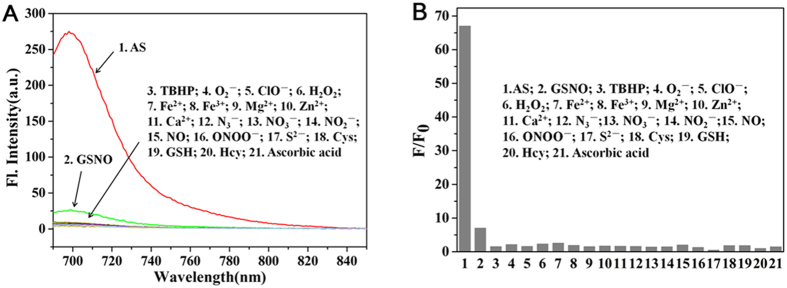
Fluorescence response of probe 1 (10 μM) after 30 min of incubation with various analytes: 1. AS; 2. GSNO; 3. TBHP; 4. KO_2_; 5. ClO^−^; 6. H_2_O_2_; 7. Fe^2+^; 8. Fe^3+^; 9. Mg^2+^; 10. Zn^2+^; 11. Ca^2+^; 12. N_3_^−^; 13. NO_3_^−^; 14. NO_2_^−^; 15. NO; 16. ONOO^−^; 17. S^2−^; 18. Cys; 19. GSH 20. Hcy; 21. Ascorbic acid. ([AS] = 100 μM; [analytes] = 200 μM).

In the next step, the selectivity of the probe towards HNO was investigated (Fig. 5). The fluorescent response of the probe towards HNO was compared with that of the probe towards various biologically relevant analytes including reactive oxygen species (TBHP, H_2_O_2_, O_2_^−^, ClO^−^), reactive nitrogen species (N_3_^−^, NO_3_^−^, NO_2_^−^, ONOO^−^, NO), reactive sulfur species (S^2−^, Cys, GSH, Hcy, GSNO), ascorbic acid and biologically related metal ions (Fe^2+^, Fe^3+^, Mg^2+^, Zn^2+^, Ca^2+^). After 30 minutes of incubation, only the addition of 200 μM of S-nitrosoglutathione (GSNO) induced a measureable probe fluorescence (7-fold enhancement). However, such an increase is considered minor when compared with the 67-fold enhancement observed with the addition of HNO (100 μM). It should also be noted that biologically relevant reductants, such as glutathione and ascorbic acid induced negligible fluorescence increment in comparison with HNO. Together, these results indicate that probe **1** offers good selectivity towards HNO over other tested species, and that the probe is indeed resistant to biological reductants such as ascorbic acid or glutathione.

### Detection of exogenous HNO

**Figure 6:**
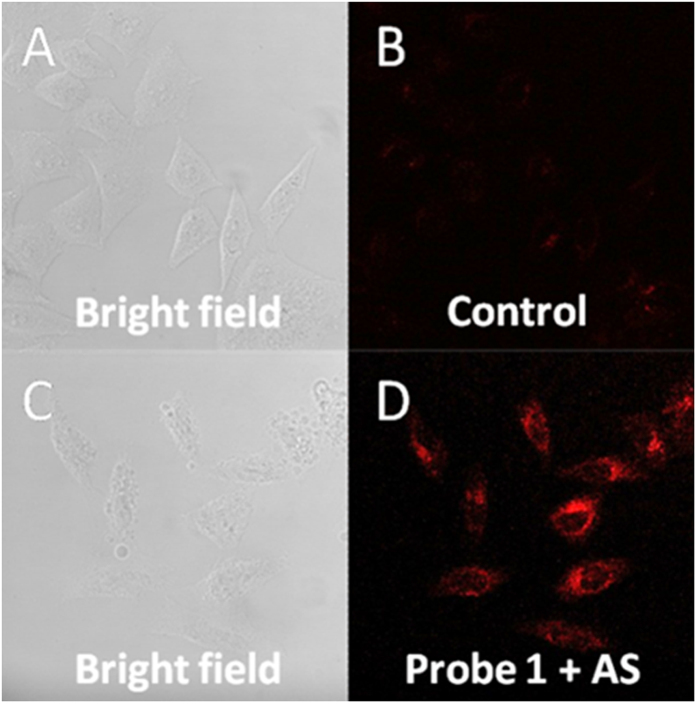
Confocal microscopy images of exogenous HNO in live HeLa cells as detected using Probe 1 (10 μM). (**A**) Bright field image of control. (**B**) Fluorescent image of control. (**C**) Bright field image of Probe **1** + AS. (**D**) Fluorescent image of Probe **1** + AS.

The probe’s ability to detect exogenous HNO in complex biological environment was tested using HeLa cells. Cells were incubated with 10 μM of probe **1** for 30 minutes and washed thoroughly to remove excess probe. AS was then added (or PBS only for control sample) and the cells were further incubated for 30 minutes. Control cells without AS showed only very weak fluorescence (Fig. 6B). In contrast, cells incubated with exogenous HNO precursor (Fig. 6D) showed a very bright fluorescence, indicating successful removal of the diphenylphosphinobenzoyl group to give strong fluorescent signal.

### Detection of endogenous HNO

Next we further investigated whether probe **1** can detect HNO formed endogenously via biological process. Recently it has been reported that NO can be reduced to HNO by biologically relevant alcohols such as ascorbate and tyrosine^38^. Additionally intracellular generation of HNO by treatment of endothelial cells with ascorbate was demonstrated by amperometric and fluorescence microscopy methods^38^. Recently Lippard’s group also carried out the imaging experiment of endogenous HNO^39^. In their experiments, it was shown that HNO could be produced endogenously by pre-treating cells with NO donor and then stimulating them with sodium ascorbate.

**Figure 7:**
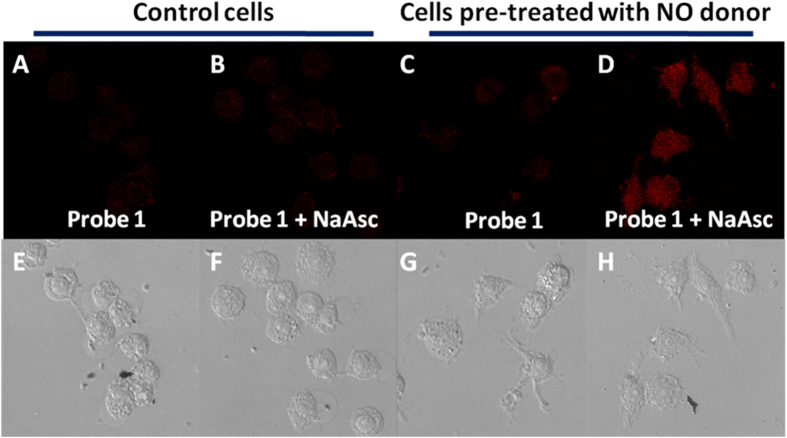
Confocal microscopy images of endogenous HNO in live RAW 264.7 macrophages using Probe 1 (10 μM). (**A,B**) are control cells whereas (**C,D**) cells were pre-treated with 200 μM of DETA NONOate for 20 h at 37 °C in DMEM. (**A,C**) cells were incubated with probe **1** for 15 min. (**B,D**) cells were further treated with 1.5 mM of sodium ascorbate for 20 minutes. (**E–H**) are the corresponding brightfield images.

In our study, we first performed the imaging experiment with RAW 264.7 cells. Addition of 1.5 mM sodium ascorbate to the control cells (without NO donor pre-treatment) showed only very weak fluorescence (Fig. 7B), whereas the addition of sodium ascorbate to the cells pretreated with DETA NONOate showed increased fluorescence signal (Fig. 7D). Similarly, fluorescence increment could be observed for HeLa cells pretreated with NO donor upon addition of ascorbate (Supplementary Fig. S8). Furthermore, the cuvette experiment with the addition of DETA NONOate and sodium ascorbate to probe **1** in PBS did not produce an increase in fluorescence (Supplementary Fig. S3), indicating that intracellular fluorescence increase is attributed to reaction with endogenously produced HNO.

### Co-localization experiment

**Figure 8:**
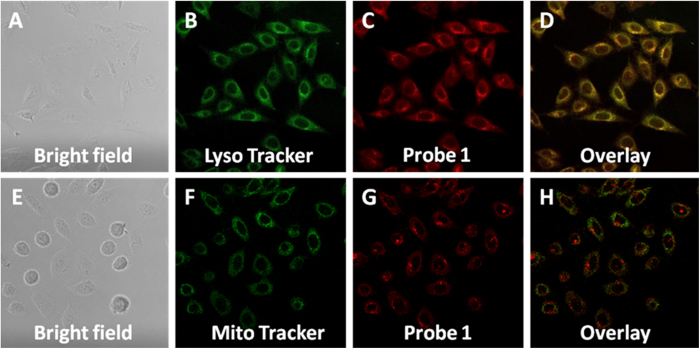
Co-localization analysis of Probe 1 with organelle-specific markers in live HeLa cells. (**A**) Bright field image. (**B**) Signal from Lyso Tracker Green. (**C**) Signal from Probe **1**. (**D**) Overlay of image of (**B,C**). (**E**) Bright field image. (**F**) Signal from Mito Tracker (rhodamine 123). (**G**) Signal from Probe **1**. (**H**) Overlay of image of (**F,G**).

Furthermore, we performed co-localization experiment to check the probe’s localization inside the cells. Experiment results revealed the majority of the probe is localized in the lysosomes (Fig. 8). The overlap coefficient with Lyso Tracker Green and Mito Tracker were calculated to be 0.81 and 0.37 respectively. The fact that probe **1** is found preferentially in the lysosomes of cells after 30 minutes indicates that it most likely undergoes endocytosis as the main way of internalization. Literature has shown that molecules that are unable to directly cross the cell membrane are usually internalized via the vesicular pathway, and later travel to endosomes before reaching the lysosomes^40^.

## Conclusion

In summary, we have designed and synthesized two new NIR probes for detecting HNO under physiological conditions. Of the two probes, probe **1** shows high sensitivity towards HNO and the detection limit was determined to be as low as 0.043 μM. The probe has excellent selectivity and was proved resistant to interferences from biological reductants. For cell imaging studies, bright fluorescence was observed from cells with the addition of exogenous HNO and also from the cells stimulated with NO donor and sodium ascorbate. Interestingly, the probe was observed to be localized mostly in lysosomes, implying that the probe might be useful for imaging HNO in lysosomes. In addition, we want to point out that the lipophilic moiety of probe **1** contains an ester bond, which can be hydrolyzed for further modification. Different localization moiety or cell-specific peptide sequences can be introduced to the probe for suborganelle or cell-specific imaging studies. We envision that the probe described here will serve as a useful tool for studying complex cellular processes involving HNO.

## Methods

### Synthesis of probes

Detailed synthesis procedure of the two probes can be found in Supporting Information. The compounds were characterized by ESI-MS, ^1^H NMR and ^13^C NMR respectively.

### Absorption and Fluorescence Measurement

Probe **1** was dissolved in an appropriate amount of DMSO to prepare 10 mM stock solution. The stock solution was then diluted in PBS buffer (10 mM, pH 7.4) to afford a final concentration of 10 μM. For absorbance studies, 10 μM probe **1** and 100 μM Angeli’s salt were mixed together, and the absorbance was measured using Shimadzu 1700 UV/vis Spectrometer. For selectivity experiments, Angeli’s salt (AS) and other biological analytes were prepared as 10 mM stock solutions in PBS buffer. Appropriate amounts of AS (100 μM) and other biological analytes (200 μM) were added to separate portions of the probe solution (10 μM) and the solution was mixed thoroughly. After 30 min of incubation, measurement of the fluorescence emission spectra was conducted using a FluoroMax-4 fluorescence photometer in a 10 mm quartz cuvette. The excitation wavelength was set at 680 nm, and the emission wavelength was set in the range of 690–850 nm. The slit widths of excitation and emission wavelength were both set at 5 nm.

### Detection limit studies

The detection limit studies were carried out following published procedures. Briefly, detection limit or limit of detection (LOD) was estimated from the mean of the blank, the standard deviation of the blank and the corresponding linear regression equation. In this manuscript, we used the following formula:


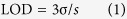



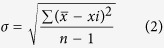


in which 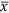 is the mean of the blank measures; xi is the values of blank measures; n is the tested number of blank measure (n = 11) and s is the slope of the linear regression equation.

### Fluorescence Microscope Experiment

HeLa cells and RAW 264.7 cells were cultured in Dulbecco’s modified Eagle’s medium (DMEM) supplemented with 10% fetal bovine serum and appropriate amounts of penicillin and streptomycin. For the microscopy experiment, approximately 1 × 10^5^ cells were seeded in confocal dishes (20 mm) with 2 mL of medium at 37 °C. Before adding the probe, the cells were allowed to adhere to the dish for 1 day. The culture medium was removed and the cells washed with DMEM once. The cells were then incubated with probe **1** (10 μM) at 37 °C for 30 min and washed twice with PBS to remove excess probe in the medium. Subsequently, a solution of 1 mM AS in NEM (N-Ethylmaleimide) treated DMEM was added to the dish and incubated for another 30 min (previous studies have shown that HNO can react with thiols in DMEM. NEM was therefore added to deplete the thiols in DMEM medium). After incubation, the cells were washed with DMEM before imaging. Fluorescence images were taken using a Leica TCS SP5 Confocal Scanning Microscope using 633 nm as the excitation wavelength and 655 to 800 nm as the emission wavelength. For the co-localization experiments, Lyso Tracker Green DND-26 and rhodamine 123 were used for the staining of lysosomes and mitochondria respectively. Lyso Tracker Green DND-26 was imaged using 488 nm as the excitation wavelength and 500 to 550 nm as the emission wavelength. Rhodamine 123 was imaged using 488 nm as the excitation wavelength and 500 to 600 nm as the emission wavelength.

### Endogenous HNO imaging experiment

Approximately 1 × 10^4^ cells were seeded in 8-wells ibidi plates, allowed to attach for 4 hours, and pre-incubated (or not for control cells) with DETA NONOate (final concentration 200 μM) for 20 h in DMEM, then washed with PBS. In order to avoid interference from free thiols in DMEM, the cells were pre-incubated in media containing N-ethylmaleimide (final concentration 1 mM) for 45 min. After the media was removed and the cells were washed with PBS once, the cells were imaged in PBS before being treated with probe **1** (final concentration 10 μM) for 15 min. The cells were then washed with PBS and another set of pictures was taken at this point. Then, a concentrated solution of sodium L-ascorbate (final concentration = 1.5 mM) was added to the wells and incubated at 37 °C for 20 min. Fluorescence images were then taken using a Leica TCS SP5 Confocal Scanning Microscope using 633 nm as the excitation wavelength and 655 to 800 nm as the emission wavelength.

## Additional Information

**How to cite this article**: Tan, Y. *et al.* Design and Synthesis of Near-infrared Fluorescent Probes for Imaging of Biological Nitroxyl. *Sci. Rep.* **5**, 16979; doi: 10.1038/srep16979 (2015).

## Supplementary Material

Supplementary Information
