# Supplementary material for: Design and Synthesis of Near-infrared Fluorescent Probes for Imaging of Biological Nitroxyl
Source: Sci Rep. 2015 Nov 20;5:16979. doi: 10.1038/srep16979 (PMC4653807; doi:10.1038/srep16979)
Supplement: Supplementary Information [file srep16979-s1.doc]

**Supporting Information**

# Design and Synthesis of Near-infrared Fluorescent Probes for Imaging of Biological Nitroxyl

Yi Tan^1,2†^, Ruochuan Liu^1,2†^, Huatang Zhang^1,2^, Peltier Raoul^1,2^, Yun-Wah Lam^1^, Qing Zhu^3^, Yi Hu^4^ and Hongyan Sun^1,2^*

^1^Department of Biology and Chemistry, City University of Hong Kong, 83 Tat Chee Avenue, Hong Kong, China, **Correspondence:* [*hongysun@cityu.edu.hk*](mailto:hongysun@cityu.edu.hk)

^2^Key Laboratory of Biochip Technology, Biotech and Health Centre, Shenzhen Research Institute of City University of Hong Kong, Shenzhen 518057, China

^3^Insitute of Bioengineering, Zhejiang University of Technology, Chaowang Road 18, Hangzhou 310014, China

^4^CAS Key Laboratory for Biomedical Effects of Nanomaterials and Nanosafety, CAS Key Lab of Nuclear Radiation and Nuclear Energy Technology, Center for Multidisciplinary Research, Institute of High Energy Physics, Chinese Academy of Sciences (CAS), Beijing 100049, China

**1. Materials and Chemicals**

All chemicals used for synthesis were purchased from commercial suppliers and applied directly without purification. Anhydrous dichloromethane (DCM) and ethanol were purchased from commercial suppliers and used as such. Angeli’s salt (AS), DETA NONOate and S-Nitrosoglutathione (GSNO) were obtained from Cayman. All reactions that utilize air- or moisture sensitive reagents were performed in dried glassware under dry N_2_ atmosphere. All spectroscopic measurements were performed in phosphate-buffered saline (PBS; 10 mM, pH 7.4). The progress of the reaction was monitored by thin-layer chromatography (TLC; Merck 60F-254). Merck silica gel 60 (70−200 mesh) was used for general column chromatography purification purpose. ^1^H NMR, ^13^CNMR, and ^31^P NMR spectra were taken on a Bruker 300 MHz or Bruker 400 MHz NMR spectrometer. For ^1^H NMR, coupling constants (J) are reported in Hertz (Hz), and multiplicity is indicated as follows: s (singlet), d (doublet), t (triplet), m (multiplet), dd(doublet of doublets), and bs (broad singlet). Mass spectra were obtained using PC Sciex API 150EX ESI-MS system. High-resolution mass spectrometry was performed with ABI Qstar Elite Q-TOF. UV absorption spectra were obtained on Shimadzu 1700 UV/vis Spectrometer. Fluorescence spectra were acquired with a Fluoro-Max-4 fluorescence photometer. Fluorescence images were captured using a Leica TCS SP5 Confocal Scanning Microscope. pH value was recorded with a Five Easy FE20 pH meter.

The solutions of various testing species were prepared from Angeli’s salt (AS, a nitroxyl source), CaCl_2_, MgCl_2_, ZnCl_2_, FeCl_3_, FeCl_2_, NaN_3_, NaNO_2_, NaNO_3_, KO_2_, NaClO, H_2_O_2_, tert-butyl hydroperoxide (TBHP), glutathione (GSH), Cysteine (Cys), homocysteine (Hcy), ascorbic acid (AA), and Na_2_S (a hydrogen sulfide source) using double-distilled water. Nitric oxide (NO) was generated from DETA NONOate (1 mM stock solution in 10 mM NaOH solution). Peroxynitrite (ONOO^−^) solution was synthesized according to literature report. Briefly, a mixture of sodium nitrite (0.6 M) and hydrogen peroxide (0.7 M) was acidified with hydrochloric acid (0.6 M), and sodium hydroxide (1.5 M) was added within 1−2 s to make the solution alkaline. The excess hydrogen peroxide was removed by passing the solution through a short column of manganese dioxide. The resulting solution was split into small aliquots and stored at −80 °C. The aliquots were thawed immediately before use, and the concentration of peroxynitrite was determined by measuring the absorption of the solution at 302 nm. The extinction coefficient of peroxynitrite solution in 0.1 M NaOH is 1670 M^−1^ cm^−1^ at 302 nm. *C*_ONOO_^−^ = Abs_302nm_ / 1.67 (mM).

**2. Synthesis and Characterization of Compounds**

**Synthesis of compound 6, 7, 8 and 9:**

Compound **6**, **7, 8**, and **9** were prepared according the modified literature method.^1-3^

**Synthesis of compound 5:**

Pyridine (0.2 ml) was added to a solution of (E)-2-chloro-3-(hydroxymethylene)cyclohex-1-enecarbaldehyde (172.6 mg, 1 mmol) and 1-benzyl-2,3,3-trimethyl-3H-indolium bromide(760 mg, 2.2 mmol) in 15 ml ethanol. The solution was stirred for 15 h at 40 °C. Upon cooling at room temperature, the resulting green solution was diluted with 40 ml DCM and washed with water (2×40 ml). The organic phase was dried over MgSO_4_ and concentrated. The crude product was purified by flash chromatography on silica with CH_2_Cl_2_/CH_3_OH(100:1 to 10:1) as eluent to afford compound **7** as a dark green solid, 680 mg, yield: 95%. ^1^H NMR (400 MHz, CD_3_OD) δ 8.42 (d, *J* = 13.8 Hz, 2H), 7.59 (d, *J* = 7.2 Hz, 2H), 7.35 (m, 16H), 6.29 (d, *J* = 14.1 Hz, 2H), 5.45 (s, 4H), 2.54 (t, *J* = 5.6 Hz, 4H), 1.83 (d, *J* = 6.0 Hz, 2H), 1.79 (s, 12H)..ESI-MS: Calcd. for [C_44_H_44_N_2_Cl]^+^: 636.3; found : 636.2.

**Synthesis of compound 4:**

Resorcinol (124 mg, 1.15 mmol) and K_2_CO_3_ (156 mg, 1.15 mmol) were placed in a flask containing 5 ml of anhydrous CH_3_CN and the mixture was stirred for 10 min at ambient temperature under nitrogen atmosphere. Compound **7** (322 mg, 0.45 mmol) was then dissolved in 5 ml anhydrous CH_3_CN and added to the mixture, followed by heating at 50 °C for 2 h. The reaction process was monitored by TLC. After the reaction was completed, the organic solvent was removed under reduced pressure and the crude product was purified by silica gel chromatography using CH_2_Cl_2_/CH_3_OH(100:1 to 10:1) as eluent to give compound **6** as blue solid(117 mg, 48%). ^1^H NMR (400 MHz, CD_3_OD) δ 8.37 (d, *J* = 13.2 Hz, 1H), 7.69 (s, 1H), 7.53 (d, *J* = 7.6 Hz, 1H), 7.36 (dt, *J* = 21.7, 10.4 Hz, 7H), 7.25 – 7.11 (m, 2H), 6.76 (d, *J* = 8.8 Hz, 1H), 6.56 (s, 1H), 5.99 (d, *J* = 13.1 Hz, 1H), 5.30 (s, 2H), 2.79 – 2.71 (t, *J* = 5.5 Hz, 2H), 2.54 (t, *J* = 5.5 Hz, 2H), 1.98 – 1.84 (m, 2H), 1.80 (s, 6H). ESI-MS: Calcd. for [C_32_H_30_NO_2_]^+^:460.6; found : 460.4.

**Synthesis of compound 3:**

Resorcinol(138 mg, 1.25 mmol) and K_2_CO_3_ (173 mg, 1.25 mmol) were placed in a flask containing 5 ml of anhydrous CH_3_CN and the mixture was stirred for 10 min at ambient temperature under nitrogen atmosphere. Compound **3** (396 mg, 0.5 mmol) was then dissolved in 5 ml anhydrous CH_3_CN and added to the mixture, followed by heating at 50 ^0^C for 2 h. The reaction process was monitored by TLC. After the reaction was completed, the organic solvent was removed under reduced pressure and the crude product was purified by silica gel chromatography using CH_2_Cl_2_/CH_3_OH(100:1 to 10:1) as eluent to give compound **2** as blue solid(153 mg, 53%). ^1^HNMR (400 MHz, CD_3_OD) δ 8.63 (d, *J* = 14.3 Hz, 1H), 7.58 (d, *J* = 7.4 Hz, 2H), 7.51 – 7.43 (m, 2H), 7.41 – 7.27 (m, 2H), 6.85 (dd, *J* = 8.7, 2.2 Hz, 1H), 6.78 (d, *J* = 2.0 Hz, 1H), 6.37 (d, *J* = 14.3 Hz, 1H), 4.53 (t, *J* = 6.7 Hz, 2H), 4.05 (t, *J* = 6.7 Hz, 2H), 2.91 (t, *J* = 6.7 Hz, 2H), 2.86 – 2.78 (m, 2H), 2.75 (t, *J* = 6.0 Hz, 2H), 1.96 (t, *J* = 5.9 Hz, 2H), 1.80 (s, 6H), 1.53 (dd, *J* = 14.8, 6.8 Hz, 2H), 1.37 – 1.27 (m, 2H), 0.89 (t, *J* = 7.4 Hz, 3H).^13^C NMR (101 MHz, CD_3_OD) δ 177.04, 170.80, 162.95, 162.51, 154.95, 145.42, 141.62, 141.39, 135.82, 129.21, 128.70, 126.51, 126.25, 122.33, 118.09, 115.13, 114.74, 112.02, 102.59, 101.58, 64.81, 50.25, 40.44, 31.56, 30.26, 28.54, 27.10, 23.70, 20.31, 18.67, 12.58. ESI-MS: Calcd. for [C_32_H_36_NO_4_]^+^: 498.6; found : 498.5.

**Synthesis of 2:**

2-(diphenylphosphino) benoic acid (61 mg, 0.2 mmol) was dissolved in 15 ml of anhydrous DCM under nitrogen atmosphere, then, 4-(dimethylamino) pyridine (30 mg, 0.24 mmol) and 1-ethyl 3-(3-dimethylaminopropyl) carbodiimide hydrochloride (38.2 mg, 0.2 mmol) were added. The mixture was stirred at ambient temperature for 30 min. Finally, compound **4** (108 mg. 0.2 mmol) was added and the resulting mixture stirred at room temperature 12 h. After the reaction was completed, the organic solvent was removed under reduced pressure and the crude product was purified by silica gel chromatography using CH_2_Cl_2_/CH_3_OH(100:1 to 50:1) as eluent to give compound **2** (62 mg, 46%). ^1^H NMR (400 MHz, CD_3_OD) δ 8.75 (d, *J* = 15.1 Hz, 1H), 8.35 – 8.31 (m, 1H), 7.73 (dd, *J* = 5.7, 2.8 Hz, 1H), 7.65 – 7.48 (m, 7H), 7.43 (dd, *J* = 5.1, 3.3 Hz, 2H), 7.41 – 7.36 (m, 8H), 7.36 – 7.33 (m, 2H), 7.33 – 7.30 (m, 2H), 7.29 – 7.26 (m, 2H), 7.10 (d, *J* = 2.2 Hz, 1H), 7.08 – 7.02 (m, 1H), 6.92 (dd, *J* = 8.5, 2.2 Hz, 1H), 5.70 (s, 2H), 2.85 – 2.73 (m, 2H), 2.61 (t, *J* = 6.1 Hz, 2H), 1.95 – 1.89 (m, 2H), 1.88 (s, 6H).  ^31^P NMR (162 MHz, CD_3_OD)δ－4.26. ESI-MS: Calcd. for [C_51_H_43_NO_3_P]^+^: 748.7; found : 748.8.

**Synthesis of 1:**

2-(diphenylphosphino) benoic acid (61 mg, 0.2 mmol) was dissolved in 15 ml of anhydrous DCM under nitrogen atmosphere, then, 4-(dimethylamino) pyridine (30 mg, 0.24 mmol) and 1-ethyl 3-(3-dimethylaminopropyl) carbodiimide hydrochloride (38.2 mg, 0.2 mmol) were added. The mixture was stirred at ambient temperature for 30 min. Finally, compound **2** (115 mg. 0.2 mmol) was added and the resulting mixture stirred at room temperature overnight. After the reaction was completed, the organic solvent was removed under reduced pressure and the crude product was purified by silica gel chromatography using CH_2_Cl_2_/CH_3_OH(100:1 to 50:1) as eluent to give crude compound **1**(59 mg, 34%). This deep blue solid was further purified by RP-HPLC using a constant flow rate of 3 ml/min with isocratic mixture of CH_3_CN/H_2_O (75/ 25) as eluent. Using this protocol, the product was collected between 9 and 10.5 min. All equivalent fractions recovered from independent runs were combined and lyophilized to yield the final pure compound **1** as deep blue solid(26 mg, 15%). ^1^H NMR (400 MHz, CD_3_OD) δ 8.78 (d, *J* = 15.0 Hz, 1H), 8.36 – 8.24 (m, 1H), 7.72 – 7.47 (m, 8H), 7.38 (dd, *J* = 10.3, 5.7 Hz, 6H), 7.28 (dd, *J* = 10.6, 4.6 Hz, 4H), 7.22 (d, *J* = 1.4 Hz, 1H), 7.04 (dd, *J* = 6.4, 4.3 Hz, 1H), 6.92 (dd, *J* = 8.4, 2.0 Hz, 1H), 6.74 (d, *J* = 15.1 Hz, 1H), 4.72 (t, *J* = 6.6 Hz, 2H), 4.06 (t, *J* = 6.7 Hz, 2H), 3.00 (t, *J* = 6.6 Hz, 2H), 2.79 (dt, *J* = 11.8, 5.7 Hz, 4H), 2.07 – 1.91 (m, 2H), 1.84 (d, *J* = 8.7 Hz, 6H), 1.63 – 1.47 (m, 2H), 1.32 (dt, *J* = 14.9, 7.3 Hz, 2H), 0.89 (t, *J* = 7.4 Hz, 3H). ^13^C NMR (101 MHz, CD_3_OD) δ 179.27, 170.64, 164.87, 160.64, 153.04, 152.76, 146.63, 142.26, 141.37, 141.08, 137.32, 134.19, 133.81, 133.60, 132.76, 131.83, 131.18, 130.02, 128.89, 128.51, 128.10, 127.59, 122.49, 119.89, 119.08, 115.02, 112.96, 109.25, 105.46, 64.88, 51.04, 41.06, 31.65, 30.26, 28.88, 26.83, 23.66, 20.08, 18.67, 12.60. ^31^P NMR (162 MHz, CD_3_OD)δ－4.24. ESI-MS: Calcd. for [C_51_H_49_NO_5_P]^+^: 786.9; found : 786.9.

**3. Supplementary Figures**


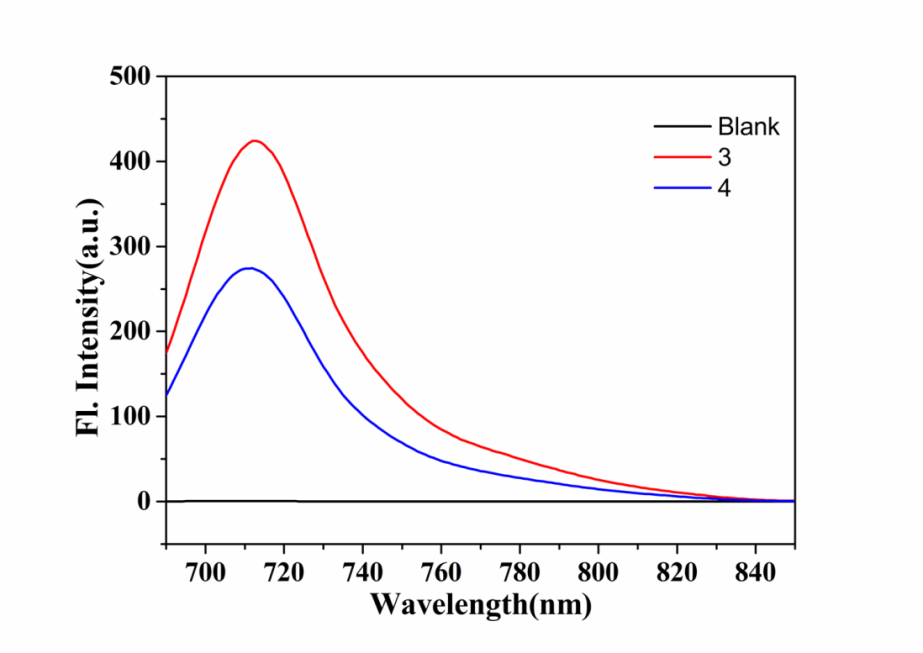


**Figure S1.** Fluorescence emission spectra of **3** (10 μM) and **4** (10 μM) in MeOH (λ_ex_ = 680 nm).


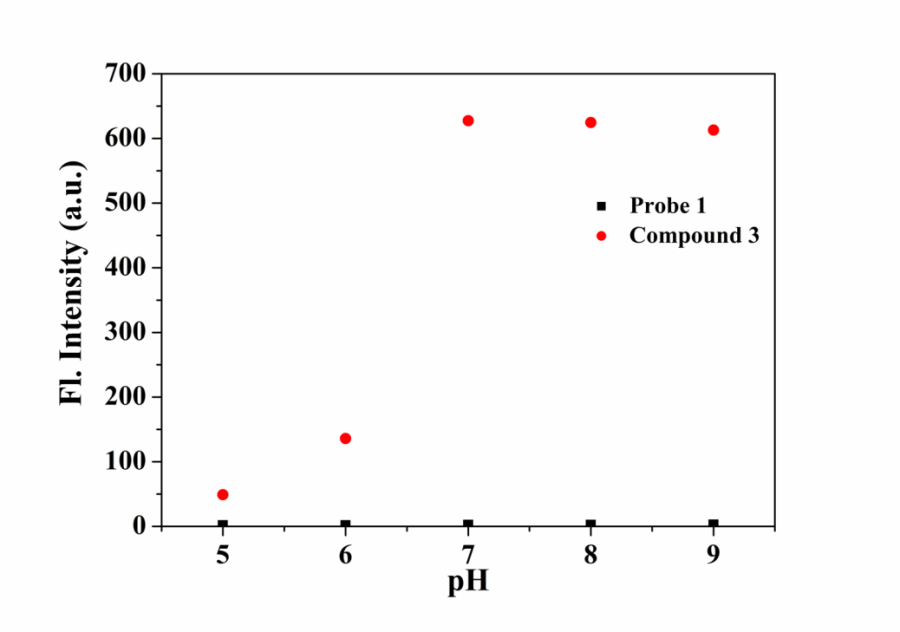


**Figure S2.** The effects of pH values on the fluorescence intensity of probe **1** (10 μM) and compound **3** (10 μM) in PBS buffer (10 mM, 1% DMSO, pH = 7.4). The experimental conditions are as follows: λ_ex_= 680 nm, λ_em_= 696 nm, pH values: 5.0, 6.0, 7.0, 8.0, 9.0.


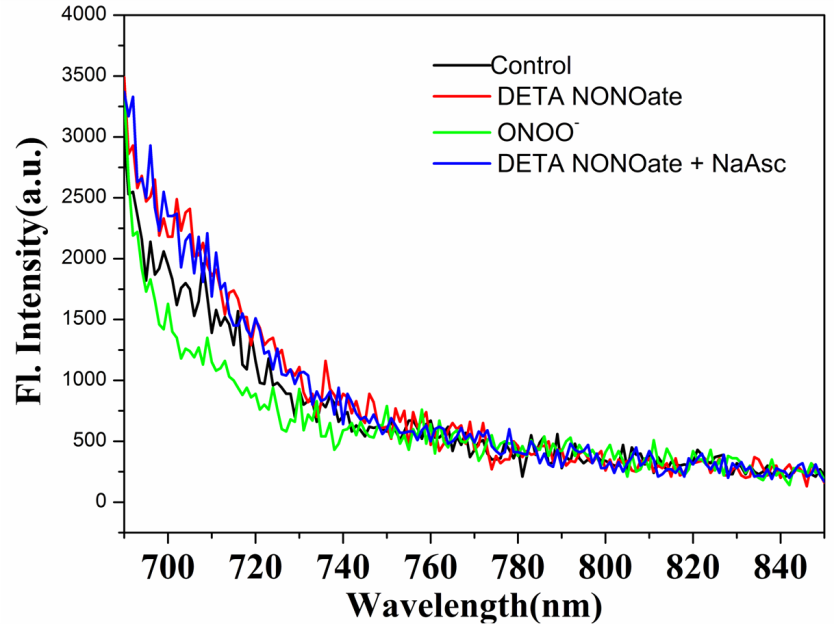


**Figure S3.** Fluorescence spectra of probe 1 (10 μM) in the presence of 200 μM of DETA NONOate, 200 μM of ONOO^-^ and 200 μM of DETA NONOate + 1.5 mM NaAsc in PBS.


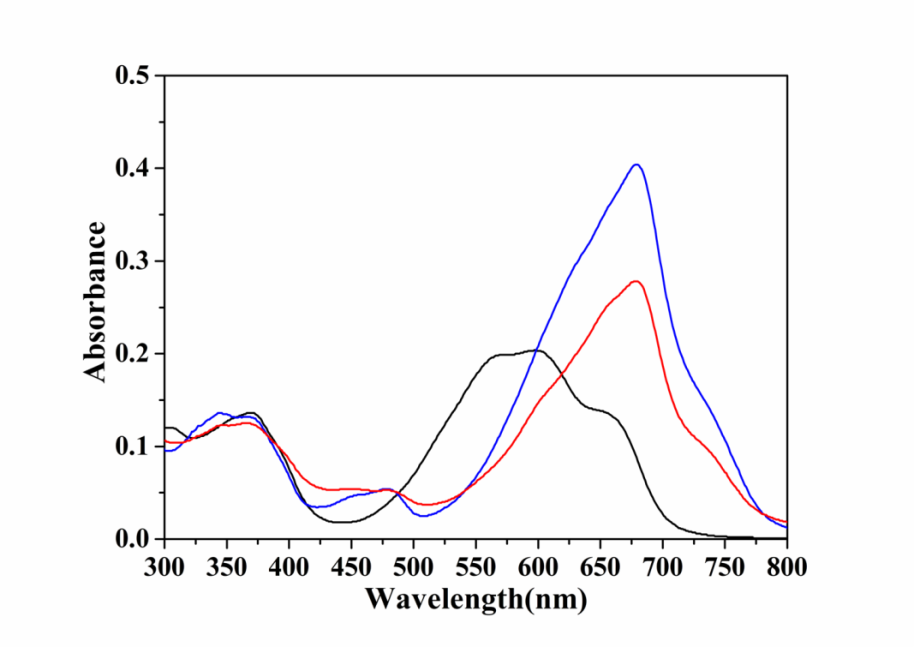


**Figure S4.** Absorption spectra of compound **1** (black line), **3** (blue line),and **1**+AS (red line). The concentrations of **1** and **3** were 10 μM and the concentration of AS was 100 μM. Incubation time was 30 min.


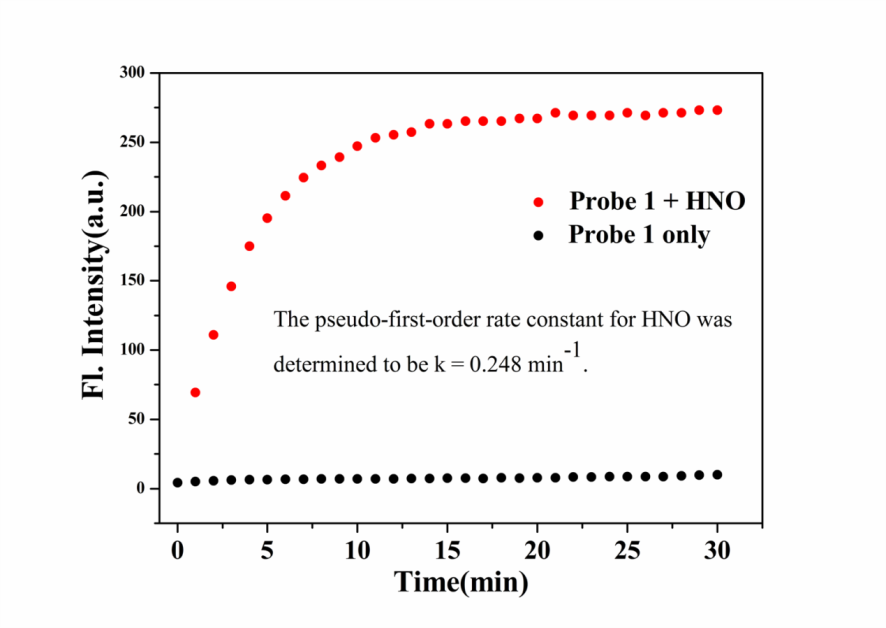


**Figure S5.** Time course experiment of Probe **1** (10 μM) reacting with AS (100 μM) in PBS buffer (10 mM, 1% DMSO, pH = 7.4).


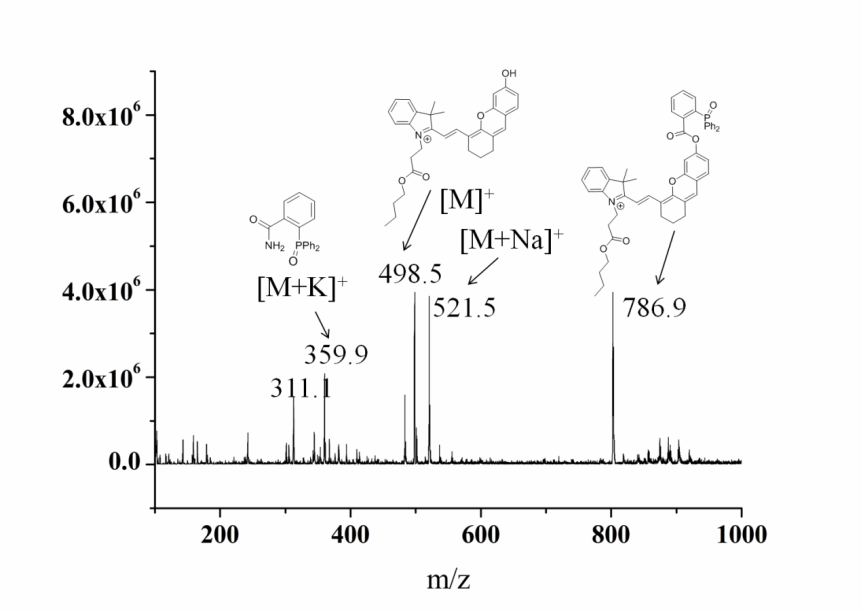


**Figure S6.** ESI-MS spectrum of **Probe 1** (0.5 mM, EtOH/PBS=1:1,1 ml) with AS (0.5 ml AS at 10 mM, 10 mM NaOH).


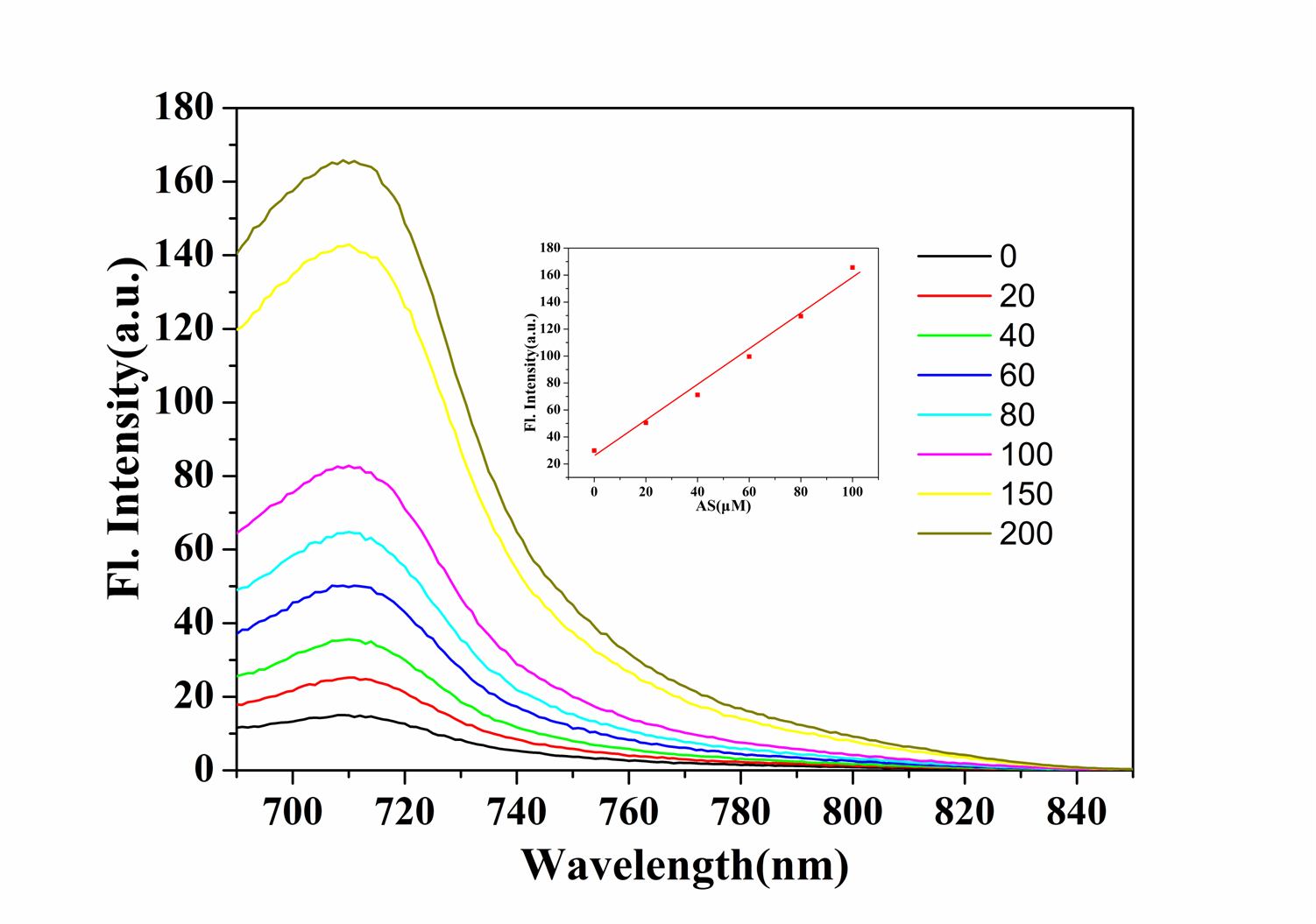


**Figure S7.** The fluorescence emission spectra of Probe 1 (10 μM) in the presence of different concentrations of AS (0, 20, 40, 60, 80, 100, 150, 200 μM) in buffered (pH 7.4) aqueous 20% bovine

serum solution.


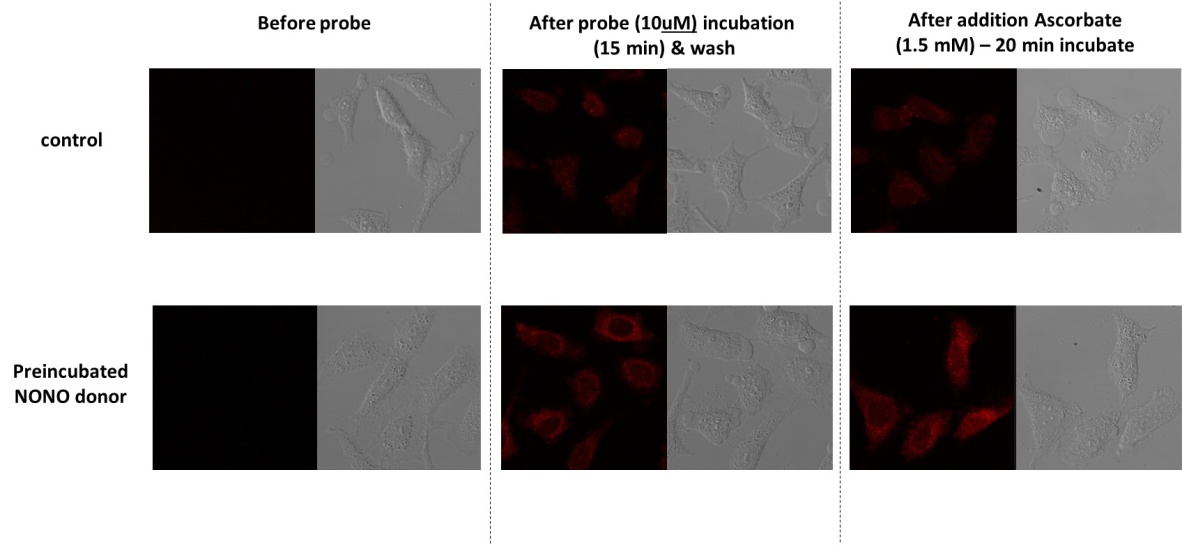
**Figure S8.** Confocal microscopy images of endogenous HNO in live HeLa cells using Probe **1** (10 µM). The images showed that, after addition of ascorbate, fluorescence increase could be observed with HeLa cells which are pretreated with NO donor, DETA NONOate.


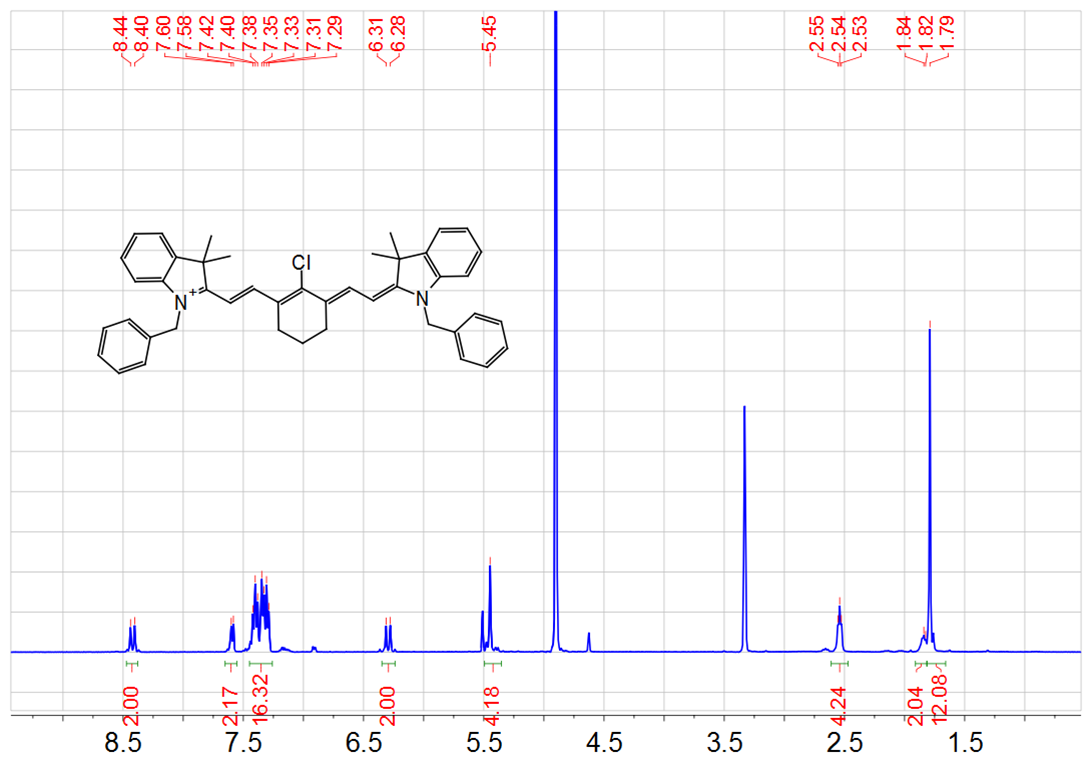


**Figure S9.** ^1^H NMR of compound **5** (400 MHz, CD_3_OD).


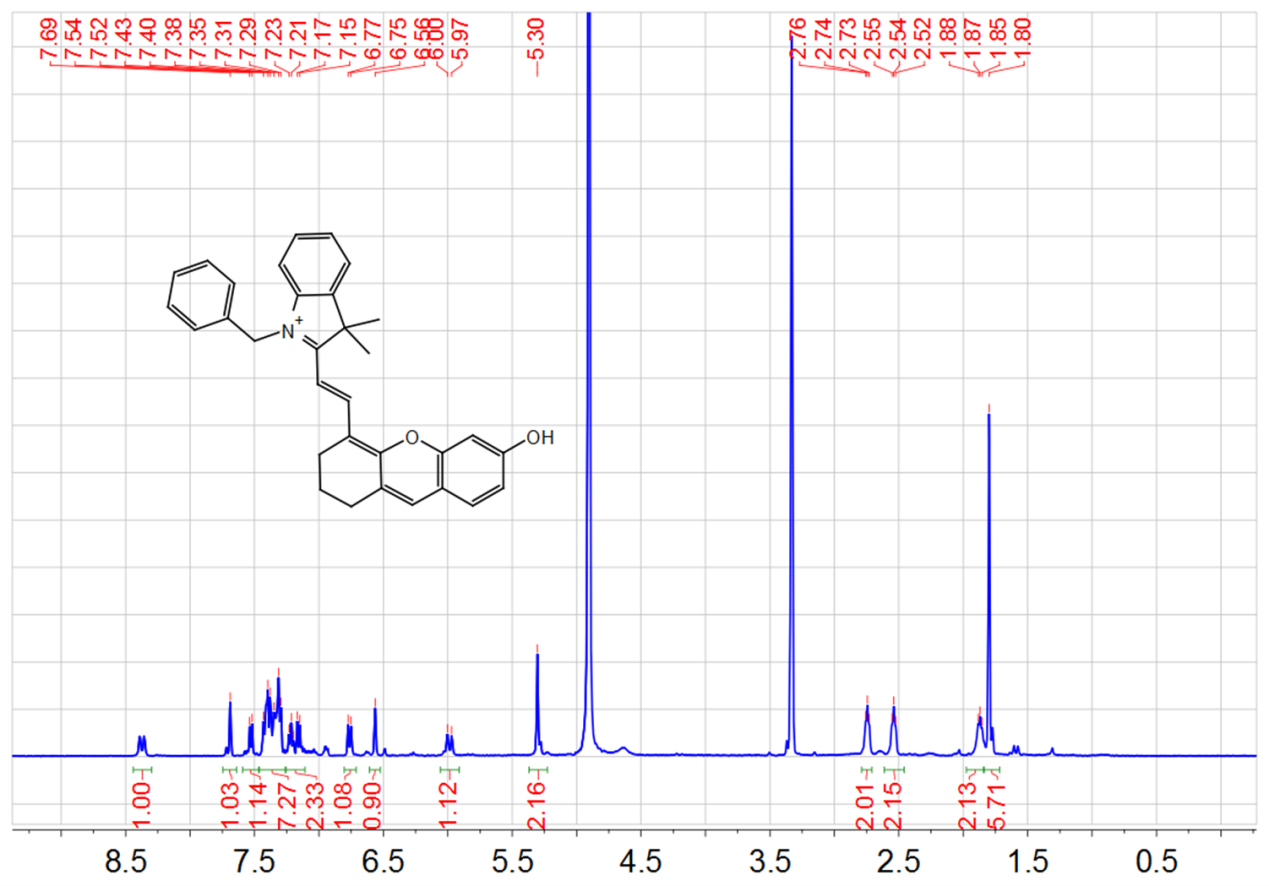


**Figure S10.** ^1^H NMR of compound **4**(400 MHz, CD_3_OD).


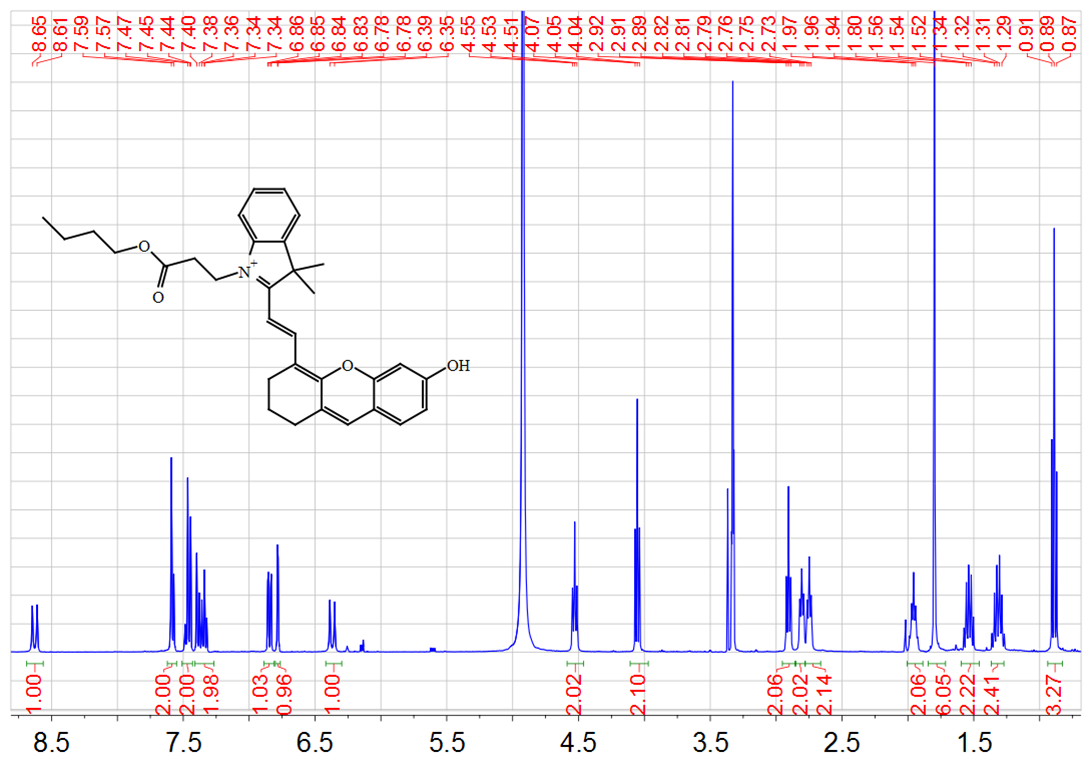


**Figure S11.** ^1^H NMR of compound **3**(400 MHz, CD_3_OD).


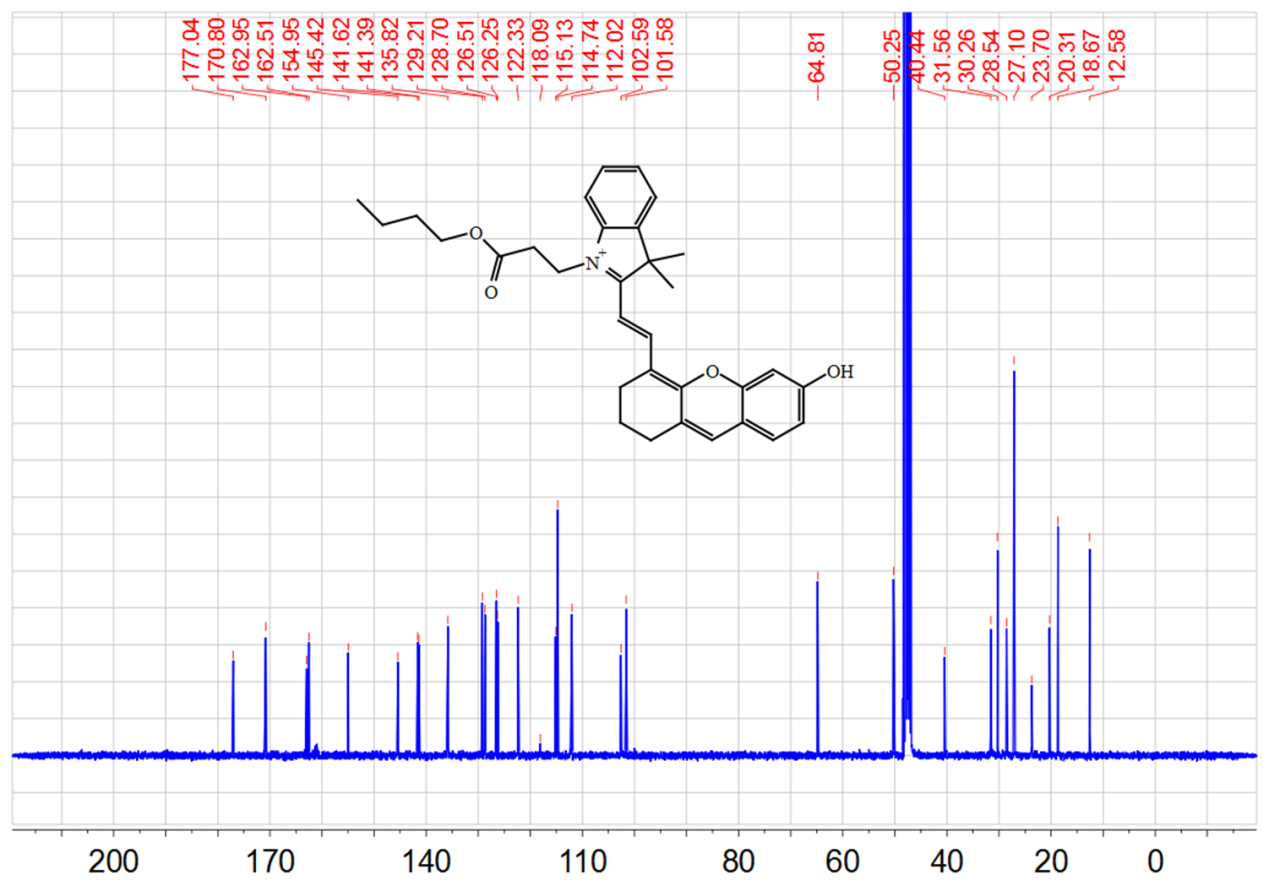


**Figure S12.** ^13^C NMR of compound **3**(101 MHz, CD_3_OD).


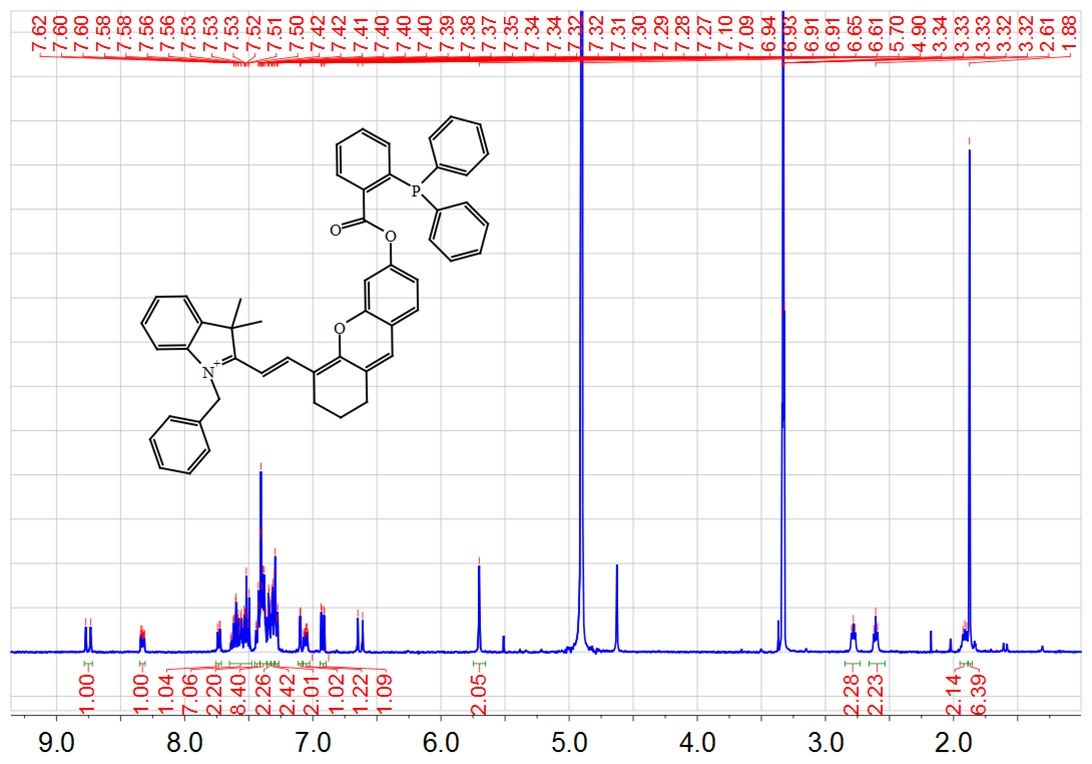


**Figure S13.** ^1^H NMR of compound **2**(400 MHz, CD_3_OD).


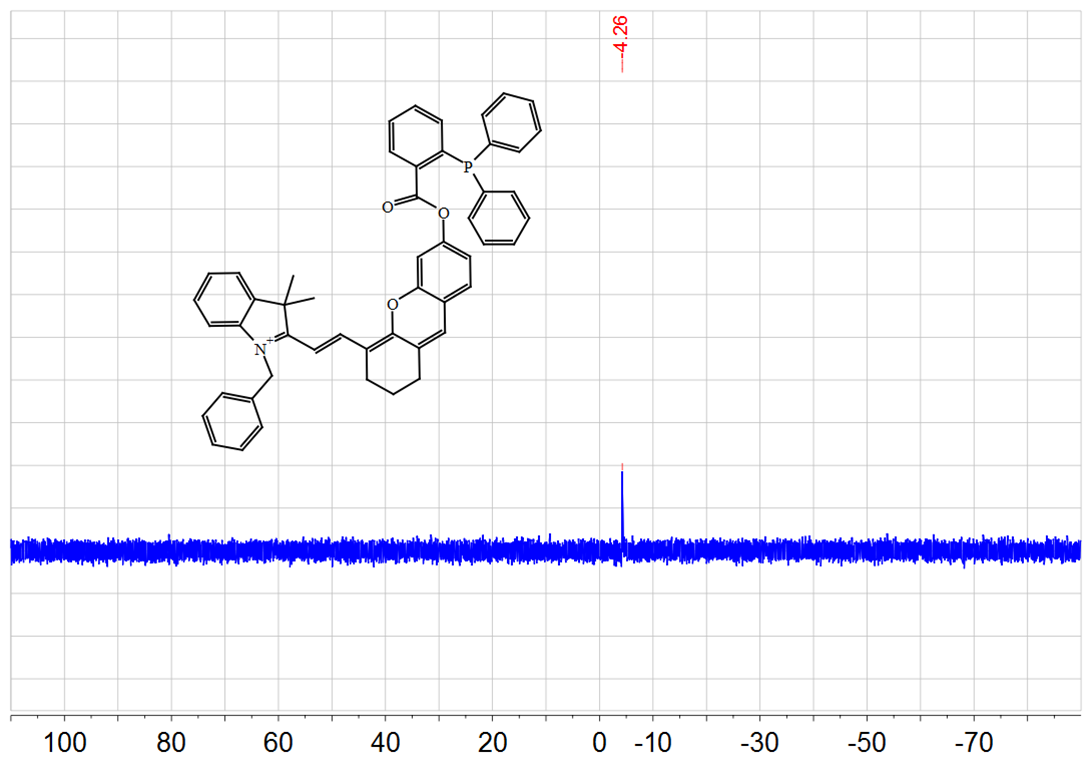


**Figure S14.** ^31^P NMR of compound **2**(400 MHz, CD_3_OD).


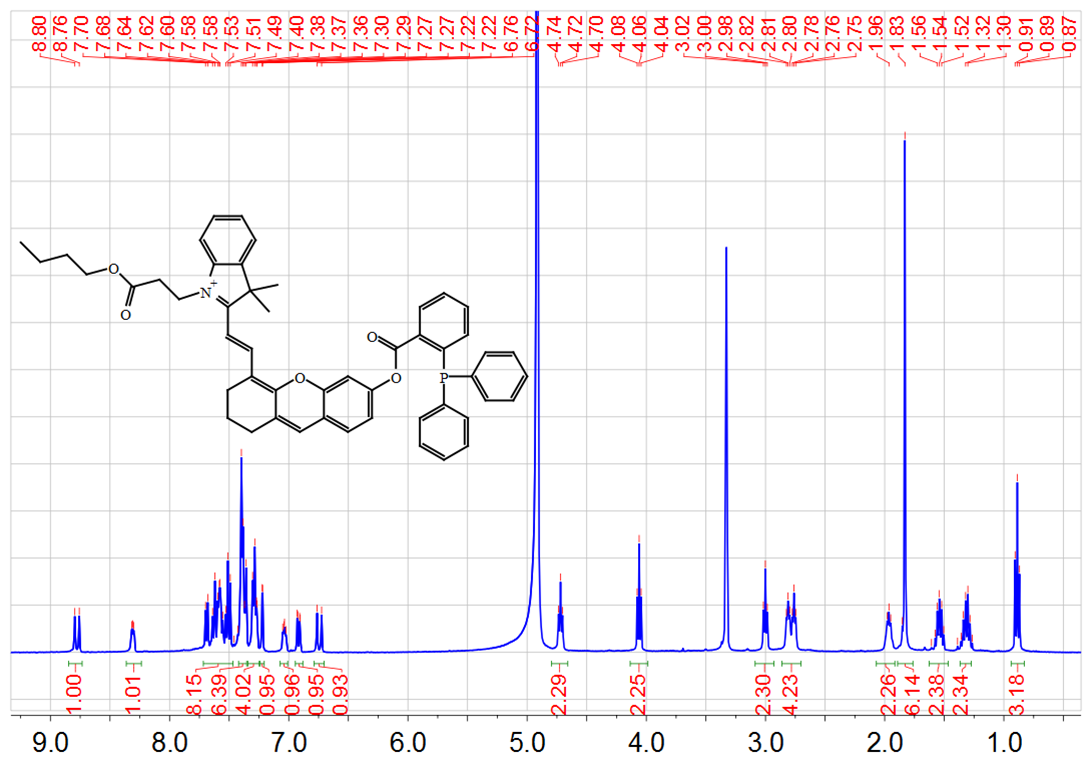


**Figure S15.** ^1^H NMR of compound **1**(400 MHz, CD_3_OD).


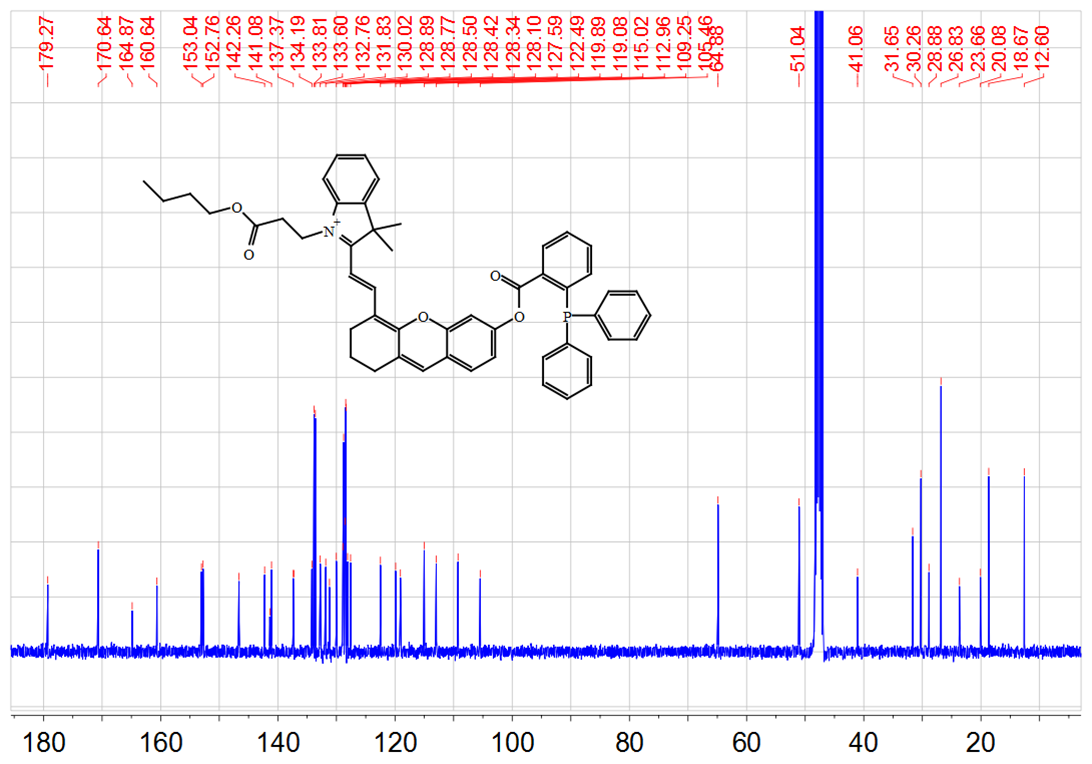


**Figure S16.** ^13^C NMR of compound **1**(101 MHz, CD_3_OD).


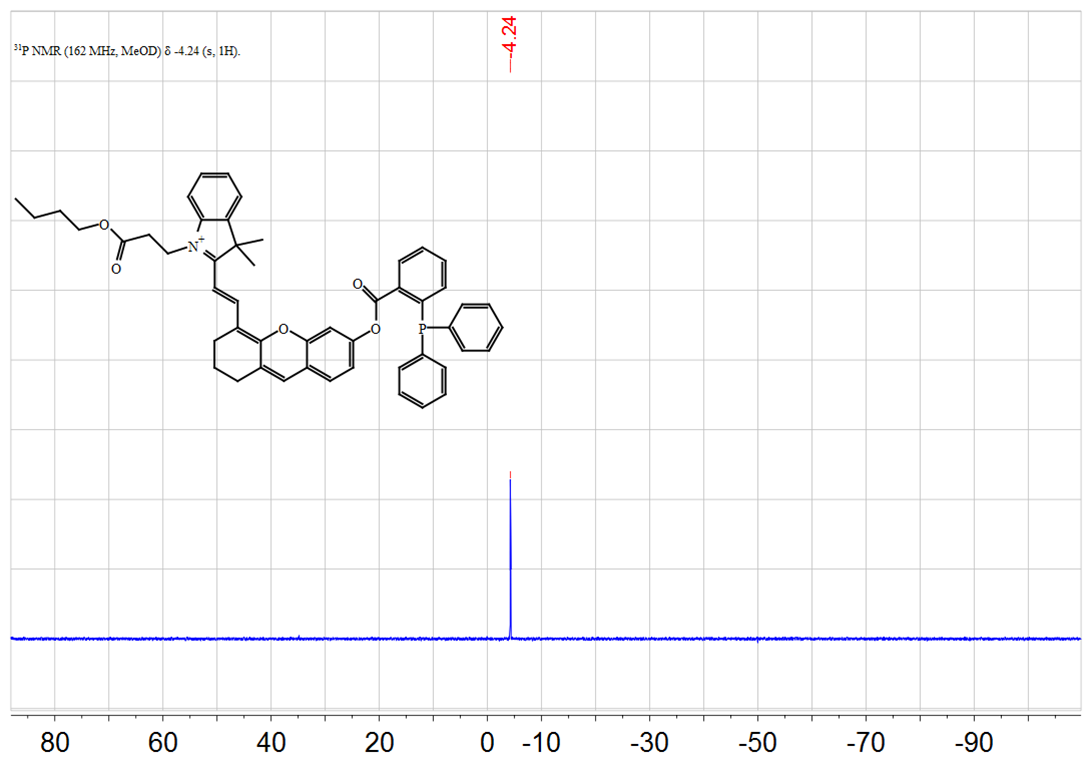


**Figure S17.** ^31^P NMR of compound **1** (162 MHz, CD_3_OD).

**Table S1. Summary of fluorescent probes for detecting HNO.**

| **References** | **Probe structure** | **Detection**  **Limit** | **Buffer** | **Application** |
| --- | --- | --- | --- | --- |
| **23** |  | ~ | 50 mM PIPES buffer (pH = 7) | Exogenous HNO imaging in cells |
| **24** |  | ~ | 50 mM PIPES buffer (pH = 7) | Exogenous HNO imaging in cells |
| **25** |  | ~ | 50 mM HEPES buffer (pH = 7.4) | Exogenous HNO imaging in cells |
| **35** |  | ~ | 50 mM PIPES buffer (pH = 7) | Exogenous HNO imaging in cells |
| **39** |  | 360 nM | 50 mM PIPES buffer (pH = 7) | Exogenous and Endogenous HNO imaging in cells |
| **28** |  | ~ | 100 mM PBS buffer (pH = 7.4) | Exogenous HNO imaging in cells |
| **29** |  | 20 nM | 100 mM PBS (pH = 7.4) | Only detect HNO in aqueous solution  and serum |
| **30** |  | 500 nM | 5 mM PBS buffer, containing 30% ethanol (pH = 7.4) | Exogenous HNO imaging in cells |
| **31** |  | 590 nM | 25 mM PBS buffer, (pH = 7.4) | Exogenous HNO imaging in cells and in a fresh rat liver slice |
| **32** |  | 1400 nM | 10 mM PBS buffer (pH = 7.4) | Exogenous HNO imaging in cells |
| **36** |  | 60 nM | 10 mM HEPES buffer containing 0.5% TW80 (pH = 5) | Exogenous HNO imaging in cells and in living mice |
| **This work** |  | 43 nM | 10 mM PBS buffer (pH = 7.4) | Exogenous and Endogenous HNO imaging in cells |

~ : not mentioned.

**4. References**

[1] Pascal,S., Haefele,A., Monnereau,C., Charaf-Eddin,A., Jacquemin,D., Le Guennic, B., Andraud,C., and Maury, O.*J. Phys. Chem. A*, **2014**, *118*, 4038–4047.

[2] Cheng,G., Fan, J., Sun,W., Cao,J., Hu,C. and Peng, X.*Chem. Commun.,***2014**, *50*, 1018-1020.

[3] Zhang, Z. and Achilefu, S. *Org. Lett.,***2004**, *6*, 2067-2070.
